# MR-guided proton therapy: a review and a preview

**DOI:** 10.1186/s13014-020-01571-x

**Published:** 2020-05-29

**Authors:** Aswin Hoffmann, Bradley Oborn, Maryam Moteabbed, Susu Yan, Thomas Bortfeld, Antje Knopf, Herman Fuchs, Dietmar Georg, Joao Seco, Maria Francesca Spadea, Oliver Jäkel, Christopher Kurz, Katia Parodi

**Affiliations:** 1 OncoRay – National Center for Radiation Research in Oncology, Faculty of Medicine and University Hospital Carl Gustav Carus, Technische Universität Dresden, Helmholtz-Zentrum Dresden-Rossendorf, Dresden, Germany; 2 Helmholtz-Zentrum Dresden—Rossendorf, Institute of Radiooncology—OncoRay, Dresden, Germany; 3 Department of Radiotherapy and Radiation Oncology, Faculty of Medicine and University Hospital Carl Gustav Carus, Technische Universität Dresden, Dresden, Germany; 4 Centre for Medical Radiation Physics, University of Wollongong, Wollongong, Australia; 5 Illawarra Cancer Care Centre, Wollongong Hospital, Wollongong, Australia; 6 Division of Radiation Biophysics, Department of Radiation Oncology, Massachusetts General Hospital and Harvard Medical School, Boston, USA; 7 Department of Radiation Oncology, University Medical Center Groningen, University of Groningen, Groningen, The Netherlands; 8 Department of Radiation Oncology, Medical University of Vienna/AKH, Vienna, Austria; 9 Christian Doppler Laboratory for Medical Radiation Research for Radiation Oncology, Medical University of Vienna, Vienna, Austria; 10 Biomedical Physics in Radiation Oncology, Deutsches Krebsforschungszentrum DKFZ, Heidelberg, Germany; 11 Department of Physics and Astronomy, Heidelberg University, Heidelberg, Germany; 12 Department of Experimental and Clinical Medicine, Magna Graecia University, Catanzaro, Italy; 13 Medical Physics in Radiation Oncology, Deutsches Krebsforschungszentrum DKFZ and Heidelberg Ion-Beam Therapy Center at the University Medical Center, Heidelberg, Germany; 14 Department of Radiation Oncology, University Hospital, LMU Munich, Munich, Germany; 15 Department of Medical Physics, Faculty of Physics, Ludwig-Maximilians-Universität München, Garching, Germany

**Keywords:** Proton therapy, Magnetic resonance imaging, Image guidance

## Abstract

**Background:**

The targeting accuracy of proton therapy (PT) for moving soft-tissue tumours is expected to greatly improve by real-time magnetic resonance imaging (MRI) guidance. The integration of MRI and PT at the treatment isocenter would offer the opportunity of combining the unparalleled soft-tissue contrast and real-time imaging capabilities of MRI with the most conformal dose distribution and best dose steering capability provided by modern PT. However, hybrid systems for MR-integrated PT (MRiPT) have not been realized so far due to a number of hitherto open technological challenges. In recent years, various research groups have started addressing these challenges and exploring the technical feasibility and clinical potential of MRiPT. The aim of this contribution is to review the different aspects of MRiPT, to report on the *status quo* and to identify important future research topics.

**Methods:**

Four aspects currently under study and their future directions are discussed: modelling and experimental investigations of electromagnetic interactions between the MRI and PT systems, integration of MRiPT workflows in clinical facilities, proton dose calculation algorithms in magnetic fields, and MRI-only based proton treatment planning approaches.

**Conclusions:**

Although MRiPT is still in its infancy, significant progress on all four aspects has been made, showing promising results that justify further efforts for research and development to be undertaken. First non-clinical research solutions have recently been realized and are being thoroughly characterized. The prospect that first prototype MRiPT systems for clinical use will likely exist within the next 5 to 10 years seems realistic, but requires significant work to be performed by collaborative efforts of research groups and industrial partners.

## Introduction

Imaging represents a key concept in the treatment planning and dose delivery workflow of contemporary radiation therapy. Image guided radiation therapy refers to the visualization and quantification of geometrical uncertainties caused by the treatment setup or changing anatomy of the patient prior to or during dose delivery.

The role of image guidance in proton therapy (PT) has been limited thus far [1]. Currently, in-room image guidance is mainly based on 2D orthogonal X-ray imaging and only in some centers in-room 3D computed tomography (CT) or on-board cone-beam CT imaging is available [2]. While the latter two are expected to reduce geometric uncertainties resulting from inter-fractional changes in patient anatomy and treatment setup, they provide poor soft-tissue contrast and have limited capabilities for intra-fractional real-time imaging. Moreover, X-ray imaging modalities deploy ionizing radiation whose exposure is associated with a long-term risk of health effects.

Precise coverage of the target volume in PT is even more challenging than in conventional photon-based radiotherapy, because protons are more sensitive to morphological variations (e.g., organ motion and deformation) and patient set-up inaccuracies than X-rays. This is due to the steep dose fall-off behind the Bragg peak and to the fact that the range of the proton beam strongly depends on the stopping power ratio (SPR) of a given tissue relative to water, which can be determined using the electron density and effective atomic number through the Bethe-Bloch equation. These uncertainties currently translate into relatively large margins (e.g. 3.5% of the range plus an additional 2–3 mm), thus compromising the dosimetric benefit of PT [3]. On top of the inherent uncertainties in predicting the SPR, errors in proton range arise from tissue density changes in the beam path due to patient setup or anatomy differences that occur between or within treatment fractions. The latter mainly relate to moving tumours and non-stationary anatomical structures that interact with the beam. This urges the need for real-time image guidance during proton beam delivery. Magnetic resonance imaging (MRI) has the ability to offer fast real-time imaging at unparalleled high soft-tissue contrast in the absence of ionizing radiation exposure. With the recent clinical implementation of real-time MR-integrated X-ray therapy (MRiXT) using MRI in combination with Cobalt-60 sources or linear accelerators as radiation device [4], there is a growing interest to investigate the concept of MR-integrated (MRiPT) as a next advancement to realize the full clinical potential of PT [5].

The integration of MRI and PT into a hybrid system should have an even higher potential to improve the targeting precision of particle therapy than for X-ray therapy. However, the following issues and open questions have to be addressed before MRiPT can be clinically implemented:

1. Mutual electromagnetic interactions between the MRI and PT system may degrade the quality of the MR image and the proton beam. The compensation of effects from the magnetic fringe fields of the cyclotron and beam line transport magnets onto the magnetic fields of the MRI scanner, as well as MR-related magnetic field effects on the beam control and monitoring systems is challenging. As data on these interferences are extremely scarce, and manufacturers of both systems typically do not specify detailed technical requirements for the combined operation of both PT and MRI systems in a particular setting, modelling and experimental investigations are required to assess the impact of these effects.
2. MRiPT requires a fast and accurate workflow for treatment monitoring, adaptation and patient-specific quality assurance (QA). Various approaches for treating patients in MRiPT are conceivable. Online adaptive replanning for stationary tumor indications (i.e. non-moving targets) would be an obvious first step to exploit the improvement offered by on-board MRI for setup verification and detection of inter-fractional changes in anatomy. The next stage would likely be adaptive replanning of moving target volumes that are treated with breath-hold gating. Ultimately, real-time MRI could assist in the management of interplay effects during full dynamic treatments with tumor tracking and rescanning under free-breathing conditions.
3. Magnetic field effects on beam transport and dose distortions of proton beams need to be taken into account for dose calculation, optimization and delivery. Several studies have investigated the proton beam transport, dosimetry changes and treatment planning in the presence of magnetic fields, in particular the Lorentz force induced beam deflection [6–14]. Fast and accurate dose calculation algorithms are required together with correction strategies to account for both the complex-shaped magnetic fringe fields and the uniform imaging field of the MR scanner in treatment planning and validation of dose measurements.
4. For online treatment planning, the dose deposition along the proton beam path needs to be calculated from MR images only. However, MR images do not contain electron density information that can be converted into SPR or water-equivalent path length information. This has driven several research groups to develop methods to convert MRI information into synthetic CT information [15–20]. For real-time MR image-guided dose delivery the conversion not only needs to be accurate but also very fast.

Each of these four aspects is further addressed in this article.

## Experimental investigations, modelling and future hardware requirements

### Experimental investigations: proof-of-concept system

MRiPT requires the operation of an MRI scanner in an environment contaminated by a transient electromagnetic field of at least two origins. Firstly, the accelerating voltage of cyclotrons and synchrotrons typically operates in the radiofrequency range of the MR scanner. Secondly, fringe fields originating from dipole and quadrupole magnets used for beam transport and focussing, respectively, as well as from fast-switching dipole magnets used for beam scanning in the nozzle could change the magnetic field homogeneity and resonance frequency during MR image acquisition, leading to severe image artifacts. Conversely, the static and dynamic MR fringe fields might impact the beam steering and control system. Ionization chambers at the beam line nozzle get exposed to the fringe fields of the MRI scanner as well as to high acoustic pressure levels generated by the MRI scanner during image acquisition. To compensate for these effects new shielding measures and image acquisition schemes might be necessary to be developed.

Recently, for the first time a proof-of-concept study was realized within a research program at OncoRay in Dresden, combining a C-shaped 0.22 T open MRI scanner with a horizontal static proton research beam line [21]. Prior to installation of the MRI scanner into the experimental room of the clinical proton therapy facility, the authors conducted a magnetic survey. Tri-axial magnetometry showed an increase in the environmental magnetic field at the beam isocenter that is in the micro-Tesla range when the beam transport magnets were energized for 70–220 MeV beams. During a full 360° gantry rotation in the adjacent treatment room, a sub-micro-Tesla change in environmental magnetic field was measured in all three field components nearby the beam isocenter. Measurements with a magnetic field camera positioned at the magnetic isocenter of the MRI scanner showed minor changes in its static magnetic field and no effects on the magnetic field homogeneity when the beam transport magnets were energized for different beam energies. From these measurements, no severe MR image degradations were expected, and hence no additional magnetic shielding was applied. First MR images of tissue-mimicking phantoms have been acquired with this in-beam MRI scanner during proton beam irradiation. No MR image degradation was observed when images were acquired during constant operation of the beam line. No visible beam-induced effects were reported.

For a more clinically realistic setting, the technical integration with a pencil beam scanning (PBS) beam line is currently under investigation, as the influence of the scanning magnets is expected to be larger than that of the beam line magnets [22]. In-silico modelling of a PBS scenario is further presented in the next section.

### Modelling of magnetic interactions between MRI scanner and PT delivery system

**Fig. 1:**
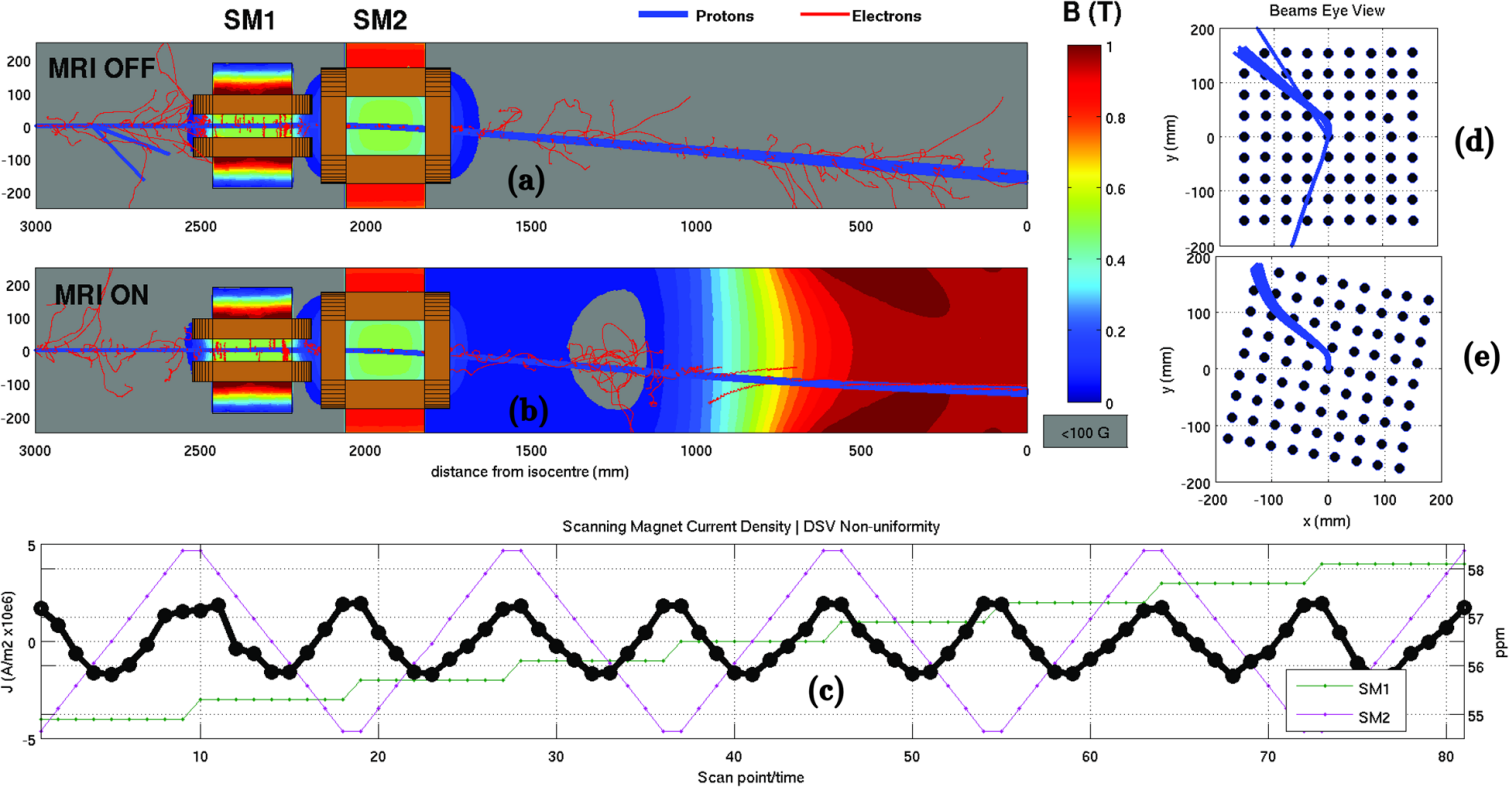
Model of the magnetic interaction between an MRI and PBS assembly when delivering a scanning pattern down the bore of the MRI. Example of a scanned pencil beam with the MRI scanner turned off (**a**) and on (**b**). **c** Scanning magnet (SM) settings and MFH over the scanning pattern shown in (**d**). **e** Changes to the scanning pattern due to the presence of the magnetic field from the MRI scanner

Finite element (magnetic field) and Monte Carlo (radiation transport) modelling methods can be used to investigate the key interactions in a potential MRiPT system. To the best of our knowledge, only one such study exists in the literature [23]. This study presents as an example a model of the 1 T split-bore MRI system of the Australian MRI-Linac programme and a typical PBS assembly (Ion Beam Applications SA, Louvain-la-Neuve, Belgium) used for delivering therapeutic proton beams. COMSOL Multiphysics® (COMSOL AB, Stockholm, Sweden) models the MRI system and the PBS system from first principles. The PBS system has been modelled to emulate a dynamic PBS pattern (Fig. 1). The variation in magnetic field homogeneity (MFH, in parts per million, ppm) of the imaging volume was evaluated on a 30 cm diameter spherical volume as a function of the scanning pattern. By importing the magnetic field maps into Geant4, the Monte Carlo simulations showed how the scanned pencil beam is deflected and distorted by the presence of the magnetic field.

**Fig. 2:**
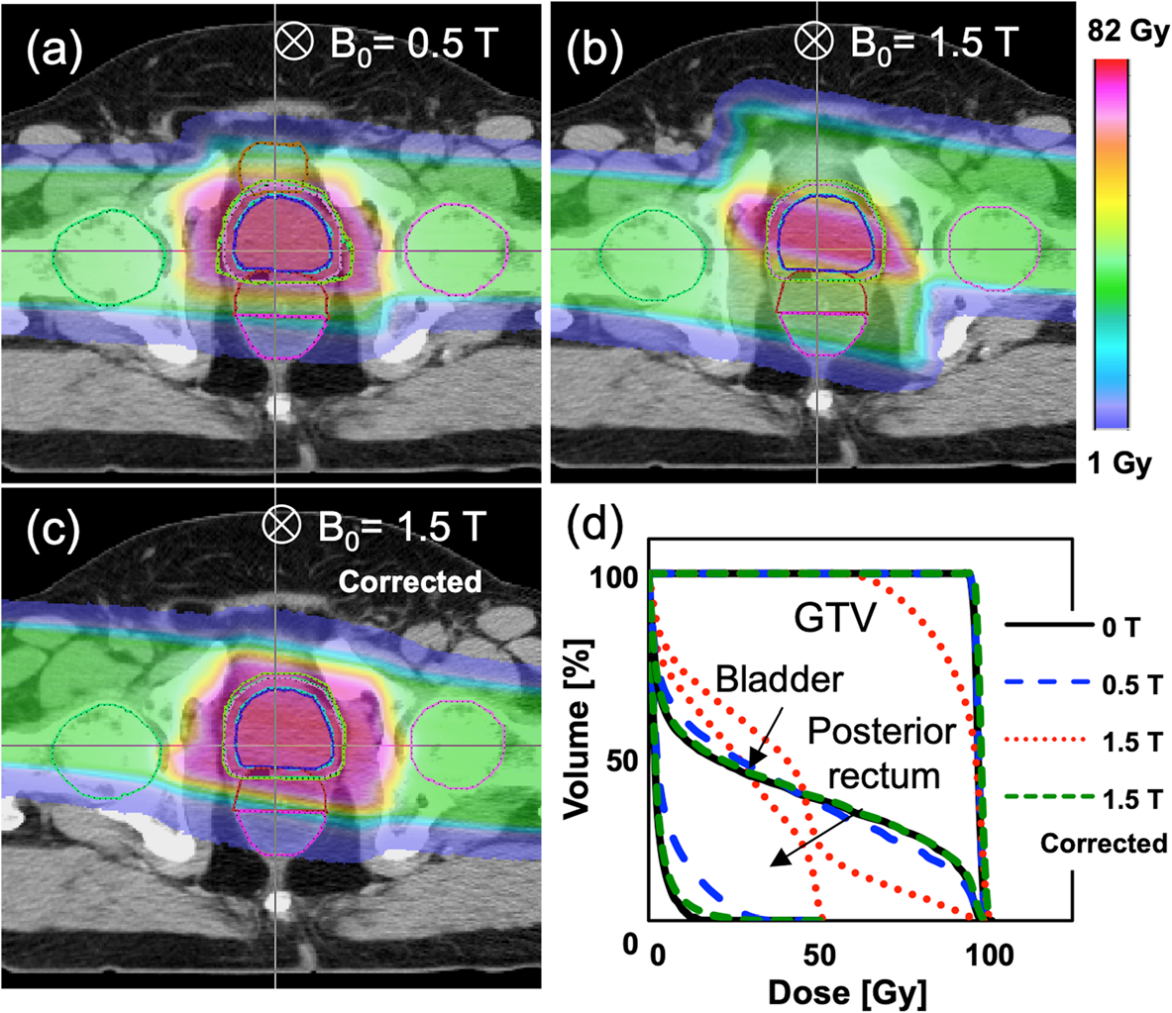
Proton dose distortions within uniform transverse magnetic field of 0.5 T (**a**), 1.5 T (**b**), and 1.5 T with delivery corrections (**c**), for a prostate plan (i.e. large range representing the worst-case scenario). **d** Dose volume histogram for the 3 above scenarios with the magnetic field on, compared to the planned dose with no magnetic field. Adapted from [8]

This integrated simulation approach allows to study (1) the impact of the PBS scanning on the MFH, and (2) the deflection of the proton trajectories as they reach the treatment volume. Typically, the MFH should be a few ppm for minimal image distortion and not change with time. The simulation results show a dynamic change in peak-to-peak MFH of < 2 ppm with an offset of 56.5 ppm. The static change to the MFH is beam energy dependent and would be correctable by passive or active shimming of the MR magnet. Experimental and Monte Carlo phantom studies have consistently shown predicable magnetic field-induced beam deflections in phantom geometries [24]. Furthermore, Monte Carlo based treatment planning studies on patients have demonstrated that dose distortions are generally negligible for magnetic field strengths up to 0.5 T (except for prostate) and correctable for up to 1.5 T (Fig. 2) [8–10]. Due to the near and far magnetic fringe fields, the proton pencil beam paths exhibit a rotation around the central beam axis. Such a change is expected to be addressed using software-based correction methods [5].

### Future hardware requirements

For MRiPT to be clinically viable, the treatment options should not be significantly compromised as compared to existing PT. In case of MRiXT, compromises were made in terms of: loss of couch angle, large couch shifts, VMAT techniques and collimator rotation. Also, with the Elekta Unity system the X-ray beam is transmitted through the aluminium cryostat of the MRI scanner, which lowers the dose rate. The following sections detail the key requirements expected in future MRiPT systems.

#### Magnet design

**Fig. 3:**
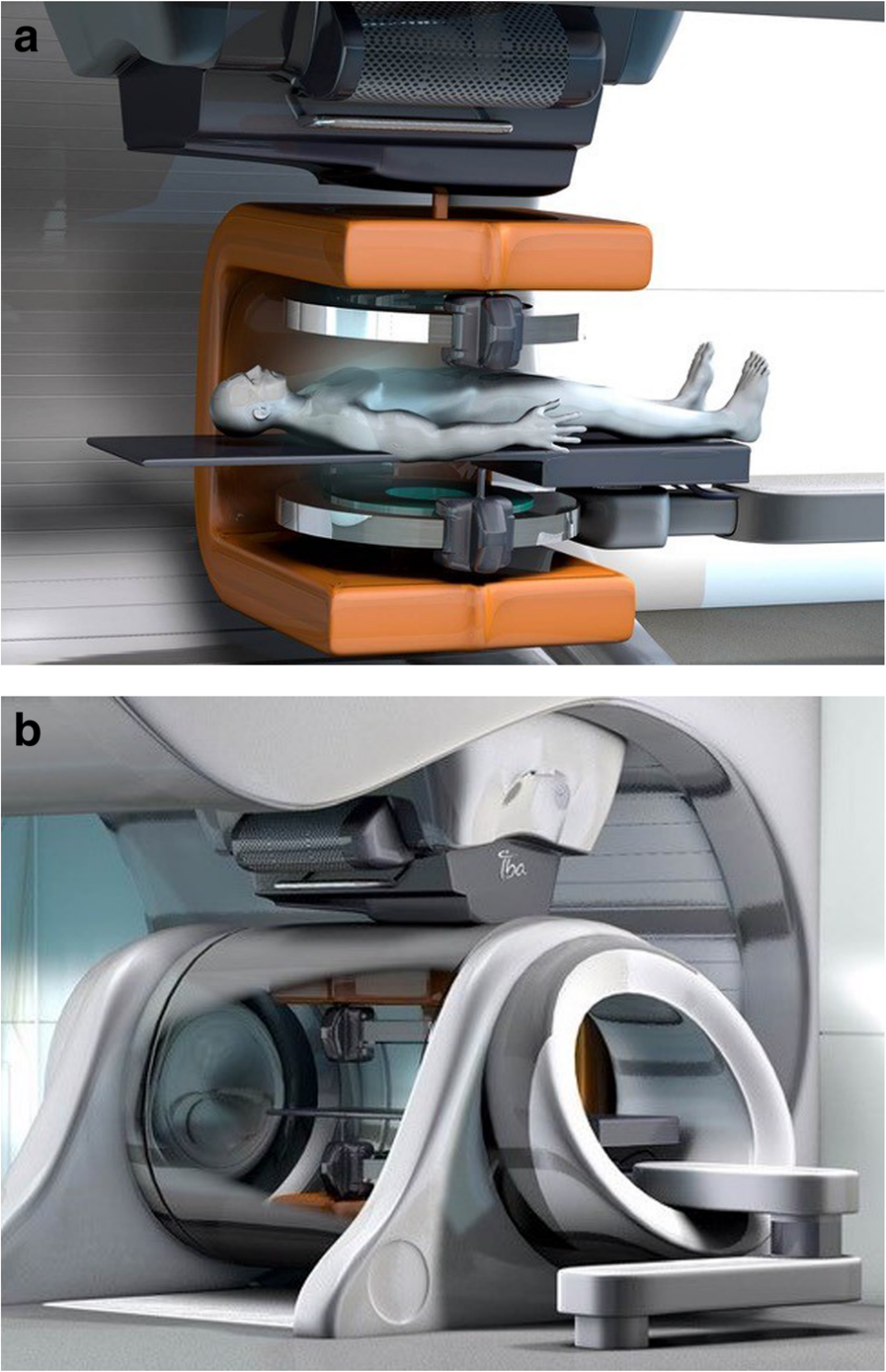
Artist impressions of (**a**) a rotating biplanar open in-beam MRI scanner integrated in a (**b**) compact proton therapy gantry treatment room (Image courtesy: Ion Beam Applications SA, Louvain-la-Neuve, Belgium)

The magnet design either needs to be open or split-bore style, such that the proton beam has direct access to the patient. For best practice, at least a partial gantry (e.g. 220° rotation) is required for beam access from any gantry angle (when coupled with a reversible patient couch). A prototype system that features an almost complete split-gap is well suited to allow a proton beam to reach the patient from all gantry angles. Here, the beam direction would be perpendicular to the main magnetic field [5]. Alternatively, an open magnet design with a C-shaped or U-shaped rotating magnet could be integrated in the proton gantry, which requires the beam to pass through one of the magnet poles (see Fig. 3).

#### MR-compatible couches

Treatment couches that are MR safe already exist in MRiXT. For MRiPT, typically couches with 6 degrees of freedom (6DOF) are used. However, such robotic couches are not MR compatible, and hence need to be modified accordingly. The 6DOF nature is highly advantageous for online adaptive therapy as a simple patient translation or rotation is performed more easily than a complete adaptive replanning based on a new patient position.

#### Gantry modifications

With MRiXT systems, the MRI scanner has been the most important structure to preserve. Adoption of the linear accelerators have been achieved through novel gantry rings that take advantage of the magnet design and housing. For MRiPT, it is expected that the integration of the MRI scanner is less obvious than for MRiXT. Magnetic decoupling of the PBS beam line from the MRI is mandatory and technically challenging. It would be natural to expect some form of additional magnetic shielding or active decoupling between the beam line and MRI. Due to the already large amount of steel components in beam lines, it could be envisaged that clever designs may utilise or modify the existing gantry structures to in fact assist or perform some of the magnetic decoupling process. Magnetic modelling will be key in ascertaining the effectiveness of any such approaches.

## Integration into clinical facilities

Before MRiPT can be integrated into clinical facilities, a number of technical aspects need to be addressed to make sure that the performance of a fully integrated system meets the clinical expectations. The entire purpose of integrated MR and PT is to be able to perform online adaptive treatments. Hence a robust workflow needs to be established which delivers that promise. In order to achieve this, fast and accurate dose calculation and planning methods need to be deployed with corresponding fast processing of the integrated MR images. Having the daily patient imaging natively as MR based introduces further complexities, in particular for PT. However an MRI-only based approach would be advantageous. The following sections describe these key elements in greater detail.

### Treatment workflow: online adaptation

Similar to the clinical introduction of MRiXT in 2014 (MRIdian by ViewRay) and 2018 (Unity by Elekta), treatment centres will be required to commence with a new and dedicated workflow that is specific to the MRiPT system. In a nutshell, the patient’s anatomy at pre-fraction imaging will be assessed and compared with the planning anatomy; the default plan will be calculated on the new patient dataset and the dose differences evaluated. If the original dose constraints are met, then treatment would continue as normal. However in the event of a failure, adaptive replanning would be invoked. For adaptive replanning, the approach is essentially the same as that for MRiXT, however several components are notably more complicated and potentially sensitive to the overall accuracy of the treatment. These include proton beam dose calculations in magnetic fields and the use of MR-only planning.

#### Plan adaptation method

The adaptation process for online adaptive treatments will be most accurately completed using full plan reoptimization. This includes calculating a new series of beamlets for the new patient anatomy and optimizing their distribution and weighting to achieve the desired dose constraints. The calculation of beamlets is however a parallelizable process as each is independent of the other, and so will benefit directly with multiple thread processing. Once generated, an optimizer will produce plans and the robustness can be assessed. More details on the calculation of proton beamlet in the context of being subject to magnetic fields are provided later on.

#### Gated treatment delivery

Gated treatments could be expected as the default treatment style. In this scenario the real-time MR information is used to confirm that the patient position has not changed significantly and that beam-on can continue as normal. For this, fast time-resolved 2D-cine MRI in a plane perpendicular to the beam direction is required [25, 26]. The MR image acquisition, reconstruction and post-processing must be fast (i.e. > 4 frames/sec) and seamlessly integrated into the software of the hybrid MRiPT system. Current MRiXT systems are reporting this process to be feasible even up to 4 frames/sec [27].

#### Dynamic treatment delivery

For tumours that move, real-time motion mitigation could be achieved through synchronizing the dynamic MR images with the beam control system to enable tumour tracking in real time. The system latency time can be minimized with the aid of motion prediction models. In the case of MRiXT, Glitzer et al. [28] describes the latencies of the MLC for real-time tracking being as small as 204 ms at 8 Hz imaging rate. For MRiPT, simple lateral tumour motion (relative the beam direction) could be in principle tracked dynamically by globally shifting the PBS delivery pattern. This would require a modified PBS controller that can superimpose a shift to the scanning currents as they are being set. However, depending on the changes of the traversed anatomy, and in the more realistic case of true 3D tumour motion, dynamic tracking may not be feasible due to the degradation of the dose coverage. This is a problem unique to PT as the Bragg peak positions depend on radiological depth, thus also requesting adjustment of the beam energy in addition to the beam lateral position. Detailed modelling and planning studies will ultimately help determine if dynamic treatments are feasible for a given patient anatomy and tumour motion.

#### Adaptive replan quality assurance

It will be a key requirement to perform an independent check of the integrity of a new adaptive patient plan. With the patient waiting on the treatment couch, this process needs to be as fast as possible. The most obvious and attractive method to achieve this will be through a dedicated independent secondary dose calculation. At present, MRiXT treatments perform this process by recalculating the dose from the new treatment plan using alternate software with different calculation algorithms. The results are compared with the primary TPS prediction and the accuracy of the match is assessed. It is highly likely that this approach will be taken with MRiPT. Careful end-to-end validation of the workflow will ensure adequate performance. Maximizing the workflow automation could lead to higher accuracy, shorter treatment times, and increased patient throughput.

Most recently, artificial intelligence (AI) has been helpful in adaptive replanning for MRiXT through an automatic MRI segmentation method using convolutional neural network (CNN) based correction networks [29, 30]. As these steps of the online adaptive workflow will be identical between MRiXT and MRiPT, we expect such AI methods to be applicable for MRiPT as well.

### Dose calculation algorithms in magnetic fields

In MRiPT, therapeutic proton beams are subject to Lorentz forces when being transported from the PBS assembly to the patient at the imaging center of the MR scanner, as well as when being transported to the treatment volume within the patient. Hence, both the non-uniform magnetic fringe field between the PBS assembly and the MR imaging center and the uniform imaging field have to be considered during the dose calculation process.

**Fig. 4:**
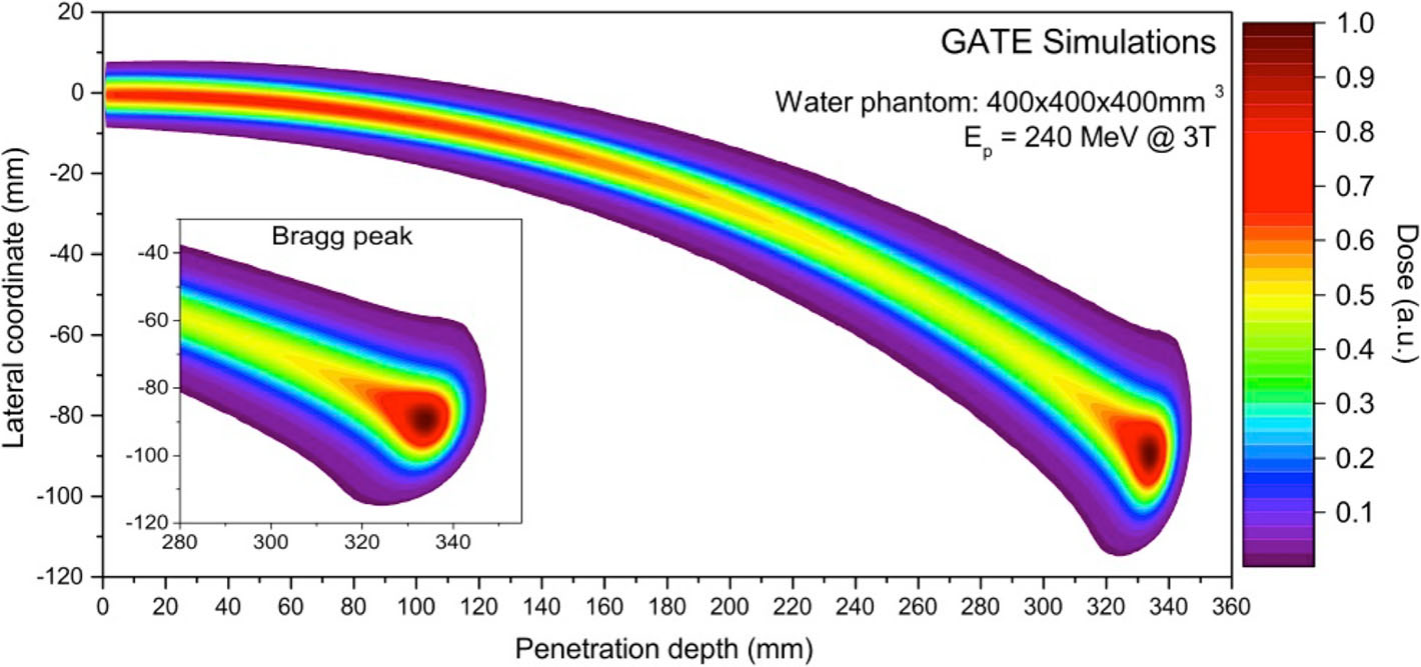
Calculated two-dimensional relative dose distribution for a 240 MeV proton beam in a homogeneous water phantom within a transverse 3 T magnetic field

The pencil beam transport within the treatment volume in the presence of a magnetic field has been simulated in different Monte Carlo environments. Generally, the magnetic forces are calculated at each step of the integration by solving the relativistic Lorentz equation for the charged particle while it loses energy during penetration. For example, in [6] and [10] Geant4 was used to investigate proton dose effects of transverse magnetic fields in water phantoms and patient geometries, respectively. In [12] GATE based on Geant4 was used to study proton and carbon ion beam transportation in magnetic fields between 0 to 3 T (Fig. 4). In [9] the TOol for PArticle Simulation (TOPAS) [31], which is also based on Geant4, was used to show the dosimetric feasibility of intensity modulated proton therapy (IMPT) plan optimization using PBS in a transverse magnetic field of 1.5 T. In [10], IMPT plan optimization using PBS in the presence of a 1.5 T magnetic field was implemented using Geant4 based dose calculations to show that the robustness of the prostate cancer treatment plans against interfractional anatomical changes and positioning errors is similar to the scenario without magnetic field in case the treatment parameters were judiciously adjusted. TOPAS was also used in a treatment planning study, which confirmed the dosimetric feasibility of MRiPT for various tumor locations using passive scattering beam delivery [8]. Although such general-purpose Monte Carlo codes deliver high-precision simulations, the main drawback is a computation time in the order of hours needed to perform simulations when low statistical uncertainty is necessary [32]. This is due to the detailed simulation of physics processes, especially the nuclear interactions [33, 34]. Meanwhile, fast Monte Carlo codes suitable for medical applications have been developed and it has been shown that this amount of details is not always necessary [35, 36]. Recently, the fast Monte Carlo software MCsquare [37] has been extended to include the effects of interactions between protons and an external uniform magnetic field. This implementation achieves computation times in the order of minutes and was experimentally validated comparing simulations and measurements of 150 MeV protons through an electro-magnet with maximum magnetic intensities from 0.5 to 1 T [38]. The speed of the dose calculation algorithm might become an essential feature in sight of real-time adaptive 4D applications foreseen for MRiPT. Final requirements for proton dose calculations in magnetic fields and MRI-only based treatment planning will depend on how MRiPT will be used clinical workflow (e.g. off-line adaptations or online daily adaptations). Corresponding requirements for treatment planning of MRgXT have been reported and discussed in [39].

Two approaches have been discussed to address the influence of the magnetic fringe field on proton pencil beam transport. One option is to use a dedicated look-up-table for a specific MRiPT design, containing (measured) deflections of pencil beams with various energies when entering the treatment volume [5]. Alternatively, the protons can be transported through a 3D magnetic field map of a specific MRiPT design, considering deflections in each transportation step, equally through the non-uniform fringe field as when entering the uniform MR imaging field. A combination of both approaches seems to be most promising: using look-up tables to account for beam offsets further away from the patient, while relying on detailed 3D magnetic field maps for the non-uniform fields close to the MR scanner and patient. Most MRiPT planning studies published so far neglect the influence of fringe fields and only investigate the impact of the imaging field component. A first planning study incorporating the fringe field component has recently been presented [40].

As mentioned above, computational speed is an essential feature for dose calculation. Despite dedicated clinical Monte Carlo dose engines for proton beam therapy, optimization and pre-calculations are often still performed with pencil beam algorithms due to their superior speed and reasonable accuracy [41]. The first pencil beam algorithms for MRiPT was based solely on an analytical approximation of the Bragg peak and beam deflection due to the magnetic field [7]. Most current pencil beam algorithms rely on look-up tables to enable a better tuning towards measurement data. Especially for larger beam deflections, the penetration depth is also affected, leading to a retraction of the Bragg peak [12, 13]. To compensate for this effect and to account for magnetic fringe fields, deflection calculation based on numerical solution of the relativistic Lorentz equation, similar to Monte Carlo codes, was integrated [12–14]. For high magnetic field strengths, depending on the energy distribution of the delivery system, additional effects, such as the deformation of the Bragg peak due to spectroscopic effects may need to be accounted for.

Validation of dose calculations in the presence of a magnetic field using a clinical TPS as well as comprehensive treatment planning studies are lacking so far. The development and validation of TPS software enabling proton beam transport through magnetic fields will follow once clinical prototypes of MRiPT units become available. The Monte Carlo solutions being introduced by vendors of TPS systems would lend themselves to be integrated with 3D magnetic field maps of MRiPT systems.

### MR-only based proton therapy planning

MRI-to-CT conversion techniques for enabling dose calculation and treatment planning can be grouped into four, often combined categories: (1) bulk density override; (2) atlas-based; (3) voxel-based and (4) deep learning techniques. Besides direct MR-to-CT conversion (MR-only) approaches, CT-to-MRI deformable image registration based approaches have been discussed [42–45]. For MR-only based photon therapy planning, extensive research has been performed and discussed in recent reviews [46, 47]. However, a limited number of contributions exist for MR-only based proton treatment planning (see Table 1), which requires a higher Hounsfield unit (HU) accuracy. One major problem is to separate and predict the correct bone and air HU values on the generated pseudo or synthetic CT (sCT) images.

**Table 1:** Overview of synthetic CT generation methods applied to patient data and evaluated for proton therapy dose calculations

Body site	Method	MRI pulse sequence	Conversion time	MAE in HU	Dose accuracy	Study
Brain	Voxel-based classification	2D TSE, 3D UTE	300 s	145	∆DVH < 2%	[15, 16]
Brain	Voxel-based classification	UTE, T_1_w	n.a.	128	∆DVH < 2%	[17]
Brain	Voxel-based classification	T_1_w, T_2_w	205 s	124	∆DVH < 0.5 Gy Γ_2%2mm_ = 98%	[20]
Brain	Deep learning	T_1_w	30 s	54	∆Range = 0.14 ± 1.11%	[48]
Brain	Deep learning	T_1_w	2 s (2D) 12 s (3D)	82–135 (2D) 82–147 (3D)	Γ_2%2mm_ = 98% (2D) Γ_2%2mm_ = 97% (3D)	[49]
Brain Prostate	Voxel-based classification	T_1_/T_2_*w dual gradient echo	30 s	42 (brain)^a^ 34 (prostate)^a^	∆DVH < 1.4% Γ_2%2mm_ = 99%	[18]
Prostate	Bulk assignment	3D dual spoiled gradient echo	120 s	83	∆DVH < 2%/2 Gy Γ_2%2mm_ = 98%	[19]
Prostate	Bulk assignment	3D dual spoiled gradient echo	120 s	81	Mean ∆DVH = 0.6%/1.5 Gy Γ_2%2mm_ = 93%	[50]
Abdomen Pediatric	Voxel-based classification	T_2_w TSE	195 s	212 (bone) 125 (lung) 52 (soft tissue)	∆DVH < 4% Γ_2%2mm_ = 88%	[51]
Liver	Deep learning	T_1_w	120 s	73	∆DVH < 1% Γ_2%2mm_ = 97%	[52]

Abbreviations: *TSE* turbo spin echo, *UTE* ultra-short echo time, *T*_*1*_*w* T_1_-weighted, *T*_*2*_*w* T_2_-weighted, *∆DVH* change in dose-volume histogram parameters, *Γ*_*2%2mm*_ 2%, 2 mm gamma criterion, *∆Range* change in proton range

^a^in pre-selected regions of interest

For this reason, first MRI-to-CT approaches used highly specialized MR sequences, such as ultra-short echo time (UTE). Rank et al. [15] employed 2D turbo spin echo (TSE) with proton density weighting and 3D UTE sequences in combination with a tissue classification approach for MRI-to-CT conversion in the brain region. Comparably large errors were observed for bone and air regions with a mean absolute error (MAE) of 145 HU. Dosimetric comparisons of proton and carbon ion treatment plans yielded deviations in the planning target volume (PTV) of 0.4–2.0%. Also Edmund et al. [17] evaluated MRI-to-CT conversion in five brain patients using two UTE (flip angles 10° and 25°) and a standard T_1_-weigthed image. Conversion was performed using six voxel-based approaches. The best results were obtained for statistical regression: MAE = 128 HU and mean error (ME) = 8 HU. Analysis of the dose-volume histogram (DVH) and the generalized equivalent uniform dose (gEUD) for tumor and brainstem showed deviations within 2%.

In contrast to these UTE based methods, Koivula et al. [18] employed a single T_1_/T_2_*-weighted dual gradient echo MRI sequence. sCT images were generated by manual delineation of bone and soft tissue on the MR images, followed by the application of two site-specific MRI-to-CT conversion models [53]. Proton dose calculation accuracy was evaluated for 10 prostate and 10 brain cases using robust planning. For a (2%, 2 mm) gamma analysis criterion (10% threshold), average pass-rates of 98.6 and 99.5% were obtained for pelvis and head. DVH parameters for the clinical target volume were within 0.6 and 1.4%, respectively. The MAEs in pre-selected volumes-of-interest were 42 HU and 34 HU. Guerreiro et al. [51] extended the methodology for application to 30 pediatric patients with abdominal tumors. A third tissue class (lung) was added and delineation of bone, soft tissue and lung was automated by atlas-based segmentation. Similar to [18], internal gas pockets were not identified on the used T_2_-weighted TSE images. For the same gamma criterion, an average pass-rate of 88.1% was reported for robustly optimized IMPT plans after body contour and internal air cavity matching with a reference CT. Differences in DVH parameters for the internal target volume were below 1%, for organ-at-risk (OAR) deviations up to 4% occurred due to considerable inter-scan differences. Average MAEs of 212 HU, 125 HU and 53 HU were reported for bone, lung and soft tissue, respectively.

A commercial certified photon-oriented sCT generation method (MRCAT [54]) was adapted and evaluated in the scope of PT by Maspero et al. [19]. The method employs a dual spoiled gradient echo sequence and Dixon reconstruction, in combination with a constrained shape bone model and bulk density assignment of 5 tissue classes for sCT generation. It was extended to allow for the identification of internal gas pockets. A MAE of 83 HU was reported. Using fully modulated IMPT plans, an average (2%, 2 mm) gamma pass-rate of 98.4% was obtained after matching of internal cavities and body outlines. DVH parameters for targets and OARs were within about 2 Gy or 2%. The median range difference for single-field-uniform-dose (SFUD) proton plans was 0.1 mm. Using the same method (without identification of internal air cavities), Depauw et al. [50] also concluded that, based on DVH parameter analysis, clinically acceptable proton dose calculation accuracy can be achieved. Also Pileggi et al. [20] performed sCT generation for 14 patients in the brain region using standard T_1_- and T_2_-weighted MRI in combination with a look-up table. Image analysis yielded an MAE of 124 HU. Median range shifts were 0.5 mm and the average (2%, 2 mm) gamma pass-rate was 98%. The worst DVH difference was 0.5 Gy.

**Fig. 5:**
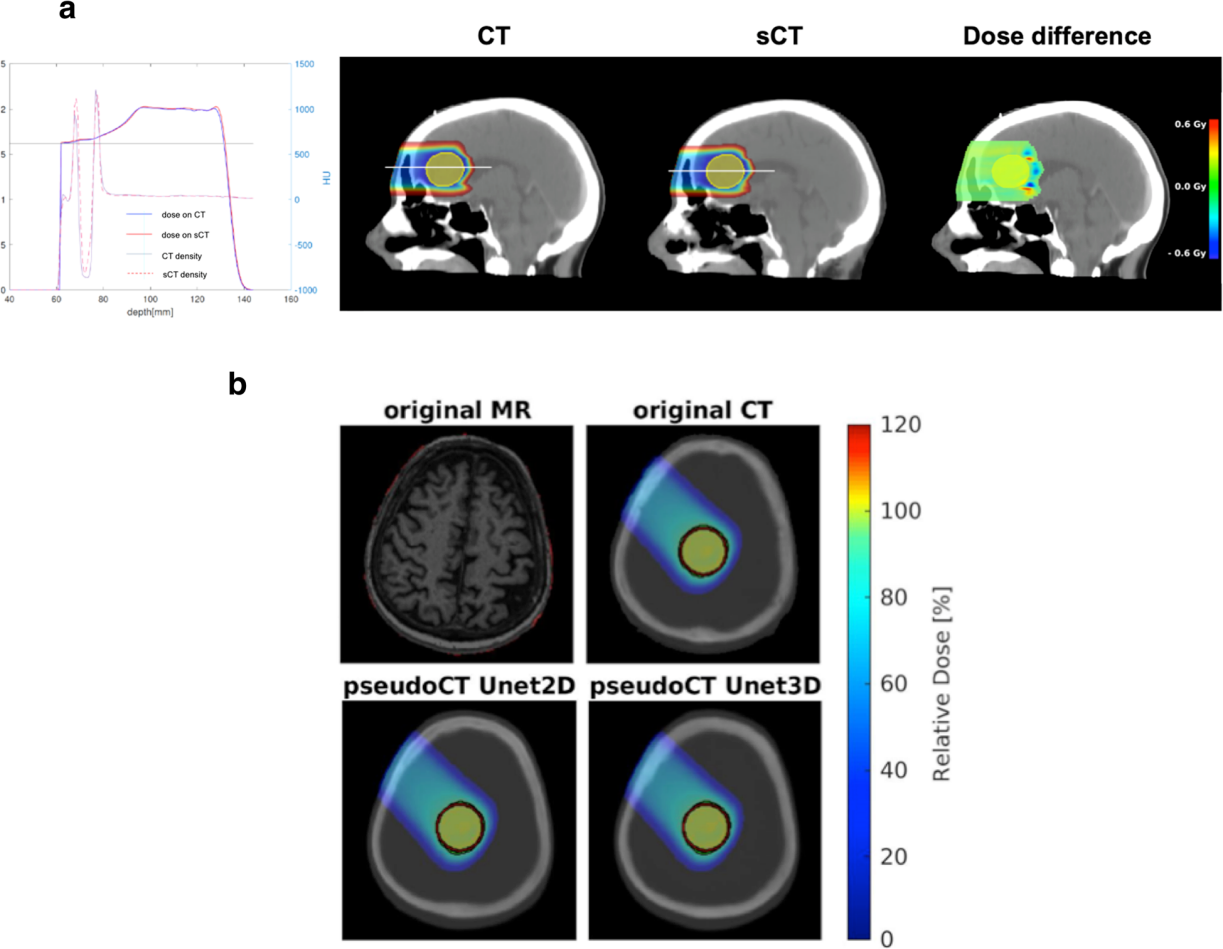
**a** From left to right: HU and dose profile of a proton spread-out Bragg peak (SOBP) for a beam entering via the frontal sinus. SOBP dose and dose difference distribution in a 2D sagittal plane as planned on the sCT and then delivered on the CT using a prescribed dose of 2 Gy. Adapted from [48]. **b** Original MRI, CT and pseudoCTs generated with a 2D and a 3D Unet for an exemplary brain case. The SFUD proton dose distribution for a single gantry angle is depicted on the original CT and the two pseudoCTs. The generic target volume is marked in red, the 95% iso-dose line in green. Adapted from [49]

In [48], for the first time a deep learning approach (U-shaped convolutional neural network [Unet]) was tested for sCT generation in PT. 15 patients with simulated tumors in the brain were planned to receive three SFUD plans (Fig. 5a). Average MAEs of 53 HU (air), 44 HU (fat), 10 HU (cerebrospinal fluid), 6 HU (white matter), 8 HU (gray matter), 119 (bone) and 54 HU (entire field of view) were determined. The relative proton range error was 0.14 ± 1.11%. In parallel, Neppl et al. [49] investigated the feasibility of utilizing deep 2D and 3D Unets for sCT generation of the head (Fig. 5b). MAE ranged from 82 to 135 HU for the 2D and from 82 to 147 HU for the 3D Unet. For SFUD proton plans, a (2%, 2 mm) gamma evaluation yielded average pass-rates of 98 and 97% for the 2D and 3D Unet. On average, more than 90% of all depth dose profiles had a range agreement better than 2 mm for both Unets. An alternative network design (3D cycle-consistent generative adversarial network) was used by Liu et al. [52] for sCT generation in the liver region using a cohort of 21 patients. An average MAE of 72.87  ±  18.16 HU was reported. In a two-proton-beam set-up, DVH deviations in the PTV below 1%, and an average (2%,2 mm) gamma pass-rate of 97% were reported. The mean range deviation was below 2 mm.

In summary, although none of the presented approaches was yet translated into the clinic in the scope of MR-only based PT, they generally showed promising results for various tumour sites, with clinically acceptable deviations from reference CT images. While initial studies focused on the use of dedicated MRI sequences for air and bone separation (e.g., UTE), more recent approaches aimed at utilizing standard MRI sequences (e.g., T_1_w) to facilitate clinical adoption. This trend could also enable usage of sequences less prone to geometric distortions (e.g., at the patient outline), which is important for accurate proton dose estimation. In terms of conversion technique, the research focus is shifting more to the application of deep learning techniques, which can theoretically handle arbitrary MRI sequences as input and provide accurate sCTs with continuous HU values in very short (~seconds) times, thus fulfilling the time requirements of MR-only workflows for online adaptive MRiPT.

## Discussion and conclusions

For moving target volumes the need for real-time image guidance in proton therapy is of even greater importance than for photon therapy. Given the successful development of different types of MR-integrated X-ray therapy devices, and the increasing clinical interest to apply them for the treatment of moving targets of soft tissues that are difficult to visualize with conventional X-ray based imaging modalities (e.g. prostate, cervix, colorectum, esophagus, liver, pancreas), it would be a logical next step to develop a similar MR-integrated concept for proton therapy. Although MRiPT is still in its infancy, several research groups have started addressing the major technical challenges and developments required for bringing this concept into clinical reality. Substantial progress has been made to uncover and understand the magnetic interactions between the MRI and PT system that affect both image and beam quality, predict and measure dosimetric effects due to the magnetic fields with high accuracy, optimize IMPT dose distributions in the presence of magnetic fields, and calculate dose distributions directly from MRI information for an MRI-only based adaptive workflow. Results from these efforts are promising and offer the prospect that the development of prototype MRiPT systems within the next 5 to 10 years should not be considered beyond the realms of possibility. To bring the MRiPT concept to the clinic, improvements in both hardware and software are required. The magnet design must be optimized for the intended treatments, and magnetic decoupling from the PBS beam line needs to be established. Fast software methods are needed for dose calculation and optimization in magnetic fields. For online adaptive replanning, fast and accurate MRI-only based treatment planning methods need to be developed and validated against current clinical standards, including the generation of reliable pseudo or synthetic CT. Here, the accuracy required for safe clinical implementation of MR-only based proton therapy planning is still debatable and might be defined individually at institutional level, e.g., by comparison of potential sCT inaccuracies to the applied clinical margins, the accepted dosimetric uncertainties, and the assumed uncertainties for CT-number to stopping power conversion. Ultimately, the methods need to be tested against the low-level uncertainty offered by proton beam range calculations based on dual-energy or proton CT images [55, 56]. Clinical adopters might have to balance the advantages of pre-treatment and/or online imaging at high soft-tissue contrast, possibly followed by treatment adaptation, against a potential loss in accuracy for stopping power prediction. This trade-off will likely depend on the treatment site, meaning that entities affected by pronounced inter- and intra-fractional changes are more likely to benefit from MRiPT. Furthermore, the development of MRI sequences allowing for the extraction of specific tissue properties (i.e., electron density, stopping power, elemental composition, water-equivalent path length) is a future research direction.

Finally, significant work needs to be performed to develop simple gantry-less prototype MRiPT systems that have the potential to be fully integrated with gantries at a later stage. This development would significantly improve the quality of proton therapy, in particular for moving tumours, and furthermore has the potential to outperform the present indications for high-precision stereotactic irradiation with MRiXT.

## Data Availability

Data sharing is not applicable to this article as no datasets were generated or analysed during the current study.

## Abbreviations

6DOF: 6 degrees of freedom
AI: Artificial intelligence
CNN: Convolutional neural network
CT: Computed tomography
DSC: Dice similarity coefficient
DVH: Dose-volume histogram
gEUD: Generalized equivalent uniform dose
GPU: Graphical processing unit
HU: Hounsfield unit
IMPT: Intensity modulated proton therapy
MAE: Mean absolute error
MFH: Magnetic field homogeneity
MR: Magnetic resonance
MRI: Magnetic resonance imaging
MRiXT: Magnetic resonance integrated X-ray therapy
MRiPT: Magnetic resonance integrated proton therapy
OAR: Organ at risk
PBS: Pencil beam scanning
ppm: Parts per million
PT: Proton therapy
PTV: Planning target volume
QA: Quality assurance
sCT: Synthetic CT
SFUD: Single field uniform dose
SPR: Stopping power ratio
TPS: Treatment planning system
TSE: Turbo spin echo
UTE: Ultra-short echo time
